# Clinical significance of programmed death 1 ligand-1 (CD274/PD-L1) and intra-tumoral CD8+ T-cell infiltration in stage II–III colorectal cancer

**DOI:** 10.1038/s41598-018-33927-5

**Published:** 2018-10-23

**Authors:** Chih-Yang Huang, Shu-Fen Chiang, Tao-Wei Ke, Tsung-Wei Chen, Ying-Shu You, William Tzu-Liang Chen, K. S. Clifford Chao

**Affiliations:** 1 Translation Research Core, China Medical University Hospital, China Medical University, Taichung, 40402 Taiwan; 2 Department of Nutrition, HungKuang University, Taichung, 43302 Taiwan; 3 Cancer Center, China Medical University Hospital, China Medical University, Taichung, 40402 Taiwan; 4 Department of Colorectal Surgery, China Medical University Hospital, China Medical University, Taichung, 40402 Taiwan; 5 Department of Pathology, China Medical University Hospital, China Medical University, Taichung, 40402 Taiwan

## Abstract

Programmed cell death-1 (PDCD1/PD-1) and its ligand programmed cell death 1 ligand 1 (CD274/PD-L1) have been reported to suppress anti-tumor T cell-mediated immune responses. However, the clinical significance of CD274 in colorectal cancer were still elusive. We aim to clarify the relationships between CD8+ intratumor-infiltrating lymphocytes (TILs) and CD274 as well as their prognostic values in stage II-III colon carcinoma. Tumor differentiation, perineural invasion (PNI), pN stage and DNA mismatch repair (MMR)-deficient were clearly correlated with CD8+ TILs counts within the tumor microenvironment (*p* < 0.0001). Furthermore, tumor differentiation and PNI were suggestively correlated with tumor CD274 expression (*p* = 0.02 and *p* = 0.0195). Tumor CD274 level was significantly correlated with higher CD8+ TILs (*p* < 0.0001) but was not associated with MMR-deficient status (*p* = 0.14). High tumor CD274 expression [hazard ratio (HR) = 2.16, 95% CI = 1.63–2.86, *p* < 0.0001] and CD8+ TILs [HR = 1.51, 95% CI = 1.19–1.91, *p* = 0.0007] were associated with improved disease-free survival and overall survival. Additionally, the subgroup of patients who had a high CD8+ TILs/tumor CD274 have better survival outcomes compared with other subgroups (71% vs 53%; *p* < 0.0001). Therefore, the CD8+ TILs counts and tumor CD274 may be prognostic factors to predict survival and therapeutic responses in stage II–III colon carcinoma patients.

## Introduction

Immune checkpoints blockades (ICB) have emerged as a promising treatment strategy and have dramatically improved long-term survival in several malignances such as melanoma^1^, non-small cell lung cancer^2^ and renal cell cancer^3^. Accumulating evidence indicates that the immune checkpoint mechanism plays an important role in suppressing tumor-specific immune responses within the tumor microenvironment. The immune checkpoint proteins programmed cell death 1 (PDCD-1/PD-1) and programmed cell death-ligand 1 (CD274/PD-L1) are expressed on both tumor cells and immune cells^4^. It is well known that the binding of CD274 (PD-L1) on tumor cells to PD-1 receptors on T cells suppresses anti-tumor T cell-mediated immune responses, inducing immunological tolerance^5^. Therefore, the complex of tumor-host interactions influences the therapeutic efficacy of these T-cell-based immunotherapies.

Colorectal cancer (CRC) is driven by genetic alterations of tumor cells and is also influenced by tumor-host interactions. Recent reports have demonstrated a direct correlation between the densities of T lymphocyte subpopulations, such as CD8+, CD45RO+ (PTPRC+), and FOXP3+ tumor-infiltrating lymphocytes (TILs), which are associated with a favorable clinical outcome in CRC, supporting a major role of T-cell-mediated immunity in repressing tumor progression of CRC. Moreover, the abundance of TILs is associated with a deficiency of DNA mismatch repair (MMR) proteins and microsatellite instability (MSI-high). Recent studies showed that microsatellite instability (MSI) and tumor-infiltrating lymphocytes (TILs) are potential biomarkers to predict the outcome of immune checkpoint blockades in CRC^6–8^. In addition, tumor CD274 upregulation within the tumor microenvironment has served as a negative feedback mechanism through CD8+ T cell infiltration and interferon-γ^4,9^ and is associated with improved survival in some malignances, such as esophageal, rectal, bladder and breast cancer^10–13^. Therefore, the complex interrelationship between tumor CD274 expression, tumor-infiltrating lymphocytes and major tumor molecular features is still elusive^7,14–16^.

In the present study, we aimed to evaluate the relationship between CD274 expression and clinicopathological characteristics, including CD8+ cell density and microsatellite instability, and the prognosis of tumors harboring CD274 expression in a large cohort of resected colon carcinomas. In addition, we aimed to analyze the long-term outcomes according to their immune status, focusing on CD274 expression and CD8+ cell density.

## Materials and Methods

### Tissue microarray construction

The TMA used for this study included tumor tissue from 911 unselected, non-consecutive and primary stage II–III colon carcinoma patients treated between 2006 and 2014 and corresponding normal mucosa specimens from China Medical University Hospital. The TMA was constructed with materials collected from the Department of Pathology and Translation Research Core. This institution performs translational research with the approval of the Institutional Review Board (IRB) in China Medical University Hospital for the use of tissue specimens [Protocol number: CMUH105-REC2-073]. The method was carried out in accordance with the committee’s approved guidelines. Construction of this TMA has been previously described in detail^17^. Briefly, areas of tumor were marked on the hematoxylin & eosin (H & E)-stained slides by pathologist. The corresponding area on the matching formalin-fixed, paraffin-embedded tissue (donor block) was then identified and marked. Tissue cylinders with a 2 mm diameter were punched from representative tissue areas of each donor tissue block and placed into one recipient paraffin blocker (AutoTiss 10 C system, EverBio Technology Inc., Taipei, Taiwan). Each TMA spot included at least 50% tumor cells.

### Immunohistochemistry

Immunohistochemistry (IHC) was performed using 3-μm-thick histological TMA sections. The antibodies used in this study were the following: anti-PD-L1 (ab205921, Abcam, Cambridge, UK), anti-MSH2 (ab92372, Abcam, Cambridge, UK), anti-MLH1 (ab92312, Abcam, Cambridge, UK), anti-MSH6 (ab92471, Abcam, Cambridge, UK), anti-PMS2 (ab110638, Abcam, Cambridge, UK) and anti-CD8 (ab4055, Abcam, Cambridge, UK). TMA slides were stained individually with horseradish peroxidase-conjugated avidin biotin complex (ABC) using a Vectastain Elite ABC Kit (Vector Laboratories, Burlingame, CA) and NovaRed chromogen (Vector Laboratories) and were counterstained in hematoxylin. Staining for CD8 was defined as positive when detected in the cytoplasm or in the cell membrane of intratumoral infiltrating lymphocytes (TILs) and was evaluated using microscopy (OLYMPUS BX53, Tokyo, Japan) according to the intensity of CD8+ TILs. Two pathologists, blind to all information about the samples, evaluated the infiltration of CD8+ TILs. With respect to the detection of CD8+ TILs, the tissue was reviewed at 40× magnification, and the area with the highest density of CD8+ TILs within the tumor microenvironment was counted at ×400 (No. of CD8+ TILs/high-power field). The average number of CD8+ TILs in five high-power fields was included in the evaluation. For CD8, a count of zero CD8+ TILs in a high-power field was assigned a score of 0, 1–3 CD8+ TILs was assigned a score of 1, 4–10 CD8+ TILs was assigned a score of 2, and >10 CD8+ TILs was assigned a score of 3. For tumor CD274 (PD-L1), tumor CD274 expression was evaluated based on immunostaining in the membrane of tumor cells according to the intensity and extent on a semiquantitative scale (0–3+) as follows: 0, absent; 1+, weak; 2+, moderate; 3+, strong membrane staining. The percentage of membranous CD274 tumor cells was recorded as follows: a score of 0 was assigned when no staining or positive tumor cell proportion was detected in <5% of the cells; a score of 1 was assigned when membranous staining was present in >5% of the positive cell proportion. The 5% threshold was based on a previous phase I trial of anti-PD-1 agents and studies for other malignancies^18,19^.

For evaluation of the DNA mismatch repair status, MMR-proficient tumors were defined as those simultaneously expressing MutL homolog 1 (MLH1), MutS homolog 2 (MSH2), MutS homolog 6 (MSH6), and PMS1 homolog 2 (PMS2), while MMR-deficient tumors were defined as those lacking expression of at least one of these markers. Based on these features, 867 colon cancers in this cohort could be classified as MMR-proficient or MMR-deficient.

### Statistical analysis

SAS statistical software, version PC 9.4 (SAS Institute, NC, USA) was used to perform the statistical analysis. All tests reported two-sided *p* values with the significance level set at 0.005^20^, and 0.005 < *p* < 0.05 labelled as suggestive evidence^20^. Student’s t test, Pearson’s chi-square and Fisher’s exact test were used to perform group comparisons. Cox regression analysis was used to estimate the hazard ratios (HRs) and 95% confidence intervals (CIs) for univariate and multivariate models. Influential factors correlated with the rectal cancer patient survival rate were adjusted in the Cox models, including sex (male versus female), age (≥65 versus <65), pT stage (tumor grade 3–4 versus tumor grade 1–2), and pN stage (positive versus negative), tumor location (proximal colon versus distal colon), CD8+ TILs (high versus low) and tumor PD-L1 (high versus low). The Kaplan-Meier estimation method assessed the five-year overall survival and disease-free survival. Survival time was defined as the time from diagnosis until relapse or death. The univariate comparison was performed using log-rank tests.

## Results

### Tumor CD274 expression and clinicopathological characteristics

**Figure 1:**
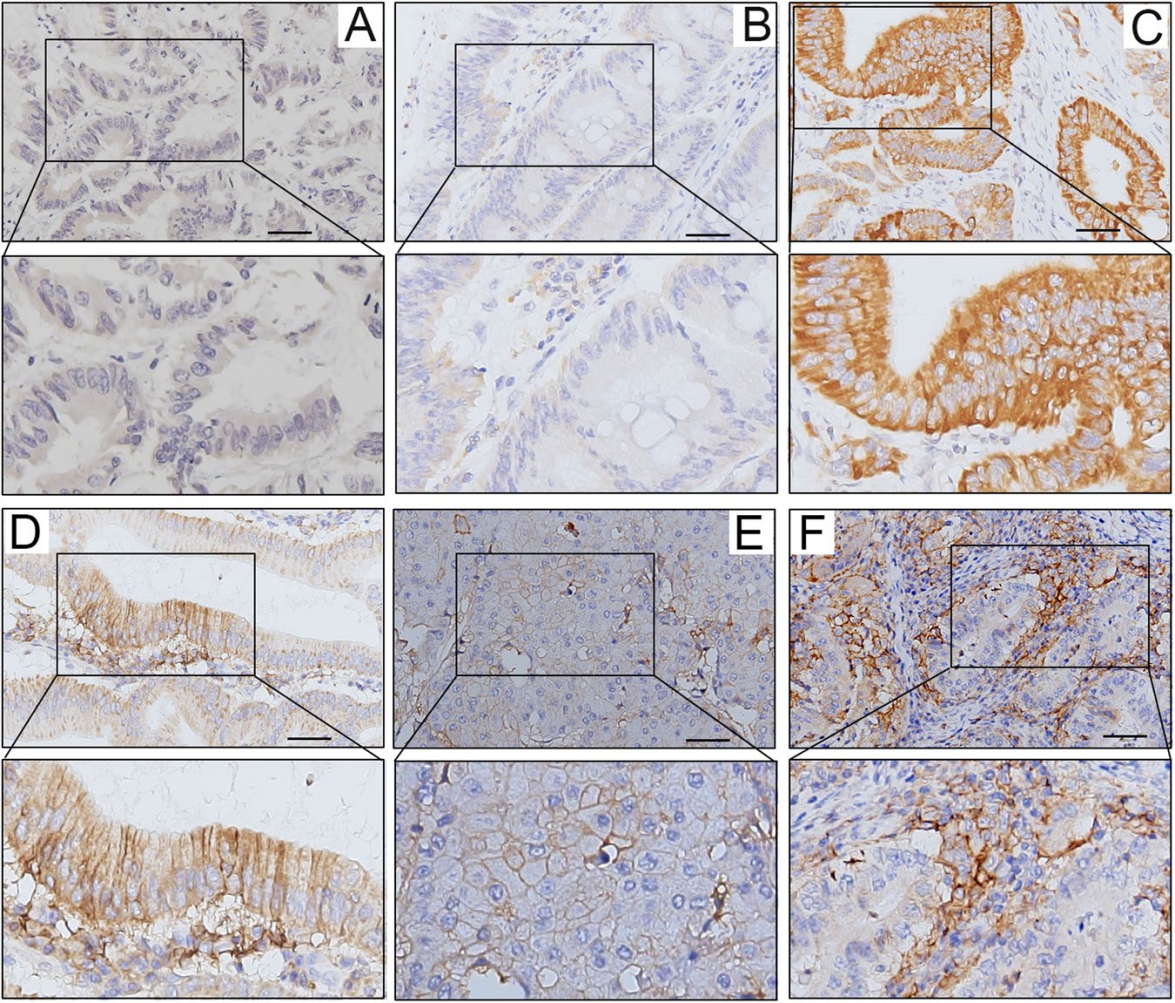
Expression patterns of tumor CD274 (PD-L1) within the tumor microenvironment of colon carcinoma. (**A**) Negative CD274 immunohistochemical staining within the tumor microenvironment. (**B**) Weak cytoplasmic CD274 expression. (**C**) Strong positive cytoplasmic CD274 expression. (**D**) Weak cytoplasmic and positive membranous CD274 expression in patients with adenocarcinoma. (**E**) Moderate positive CD274 expression with a membranous pattern. (**F**) Strong tumor membranous CD274 expression surrounding high CD274-positive inflammatory cells. Scale bar = 50 μm.

**Figure 2:**
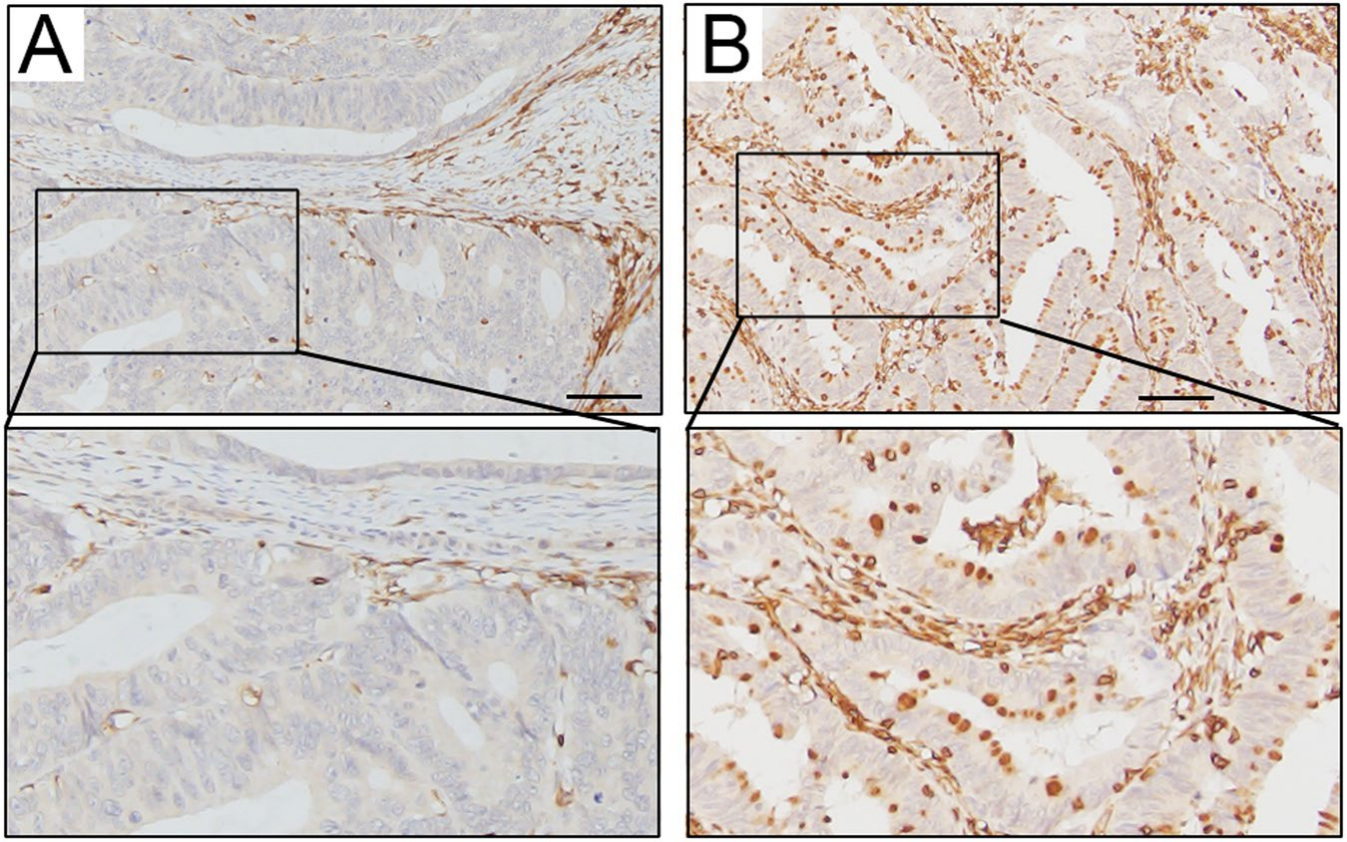
Intra-tumoral CD8+ TILs within the tumor microenvironment of colon carcinoma. (**A**) Low intra-tumoral CD8+ TILs immunohistochemical staining within the tumor microenvironment. (**B**) High intra-tumoral CD8+ TILs immunohistochemical staining within the tumor microenvironment.

We examined the expression levels of the CD274 protein in 867 cases of colon carcinoma treated at China Medical University Hospital in a retrospective cohort study. We scored tumor CD274 expression levels in the membrane (absent or present, based on the criteria of 5% tumor membranous staining) (Fig. 1). We used the sum of the cytoplasmic and membrane scores (ranging from 0 to 4) in each case for further analyses. Representative staining of the tissues under investigation, as observed upon incubation with antibodies specific for CD274 (clone 28–8 mAb), are shown in Fig. 1. CD274 was detectable in epithelial cells from normal colonic mucosa (Fig. 1A), and, importantly, in cancer cells (Fig. 1B–E). Intra-tumoral CD8+ TILs were detected within tumor microenvironment (Fig. 2)

Clinical, pathological and molecular characteristics according to the tumor CD274 expression, CD8+ intratumoral infiltrating lymphocytes (CD8+ TILs) score and the status of microsatellite instability (MSI) status in colorectal cancer are summarized in Table 1. In 867 cases of colon carcinoma, 384 patients (44%) had high tumor CD274 expression, 480 patients (56%) had low tumor CD274 expression and 4 patients had no results (tissues not available). Moreover, 283 patients (33%) were observed to have high CD8+ TILs, and 584 patients (67%) had low CD8+ TILs within the tumor microenvironment. In 867 cases of colon carcinoma, 795 MMR-proficient (92%) and 68 MMR-deficient cases (8%) were observed.

**Table 1:** Tumor Characteristics and immune status in stage II-III colon carcinoma patient.

Clinicopathological parameters	Total no.	CD8+ TILs		*p* value	Total no.	Tumor CD274/PD-L1		*p* value
		High	Low			High	Low	
	867	283 (33%)	584 (67%)		864	384 (44%)	480 (56%)	
Sex				0.39				0.7
Female	401 (46%)	125 (44%)	276 (47%)		401 (46%)	181 (47%)	220 (46%)	
Male	466 (54%)	158 (56%)	308 (53%)		463 (54%)	203 (53%)	260 (54%)	
Age				0.16				0.59
<65	387 (45%)	136 (48%)	251 (43%)		385 (45%)	175 (46%)	210 (44%)	
≥65	480 (55%)	147 (52%)	333 (57%)		479 (55%)	209 (54%)	270 (56%)	
Tumor location				0.02				0.68
Proximal colon	425 (49%)	155 (55%)	270 (46%)		423 (49%)	185 (48%)	238 (50%)	
Distal colon	442 (51%)	128 (45%)	314 (54%)		441 (51%)	199 (52%)	242 (50%)	
pT stage				<0.0001*				0.001*
Tis	2 (0.2%)	1 (0.4%)	1 (0.2%)		2 (0.2%)	1 (0.3%)	1 (0.2%)	
pT1	3 (0.4%)	0 (0%)	3 (0.5%)		3 (0.4%)	2 (0.5%)	1 (0.2%)	
pT2	29 (3%)	15 (5%)	14 (2%)		29 (3%)	15 (4%)	14 (3%)	
pT3	701 (81%)	239 (84%)	462 (79%)		698 (81%)	311 (81%)	387 (81%)	
pT4	132 (15%)	28 (10%)	104 (18%)		132(15%)	55 (14%)	77 (16%)	
pN stage				<0.0001*				0.85
Negative	457 (53%)	176 (62%)	281 (48%)		456 (53%)	204 (53%)	252 (53%)	
Positive	410 (47%)	107 (38%)	303 (52%)		408 (47%)	180 (47%)	228 (48%)	
pathological TNM stage				<0.0001*				0.85
Stage II	457 (53%)	176 (62%)	281 (48%)		456 (53%)	204 (53%)	252 (53%)	
Stage III	410 (47%)	107 (38%)	303 (52%)		408 (47%)	180 (47%)	228 (48%)	
Tumor differentiation				<0.0001*				0.02
Well to moderate	734 (85%)	217 (77%)	517 (89%)		732 (85%)	313 (82%)	419 (87%)	
Poor	121 (14%)	63 (22%)	58 (10%)		120 (14%)	65 (17%)	55 (11%)	
Unknown	12 (1%)	3 (1%)	9 (2%)		12 (1%)	6 (2%)	6 (1%)	
Lymphovascular invasion (LVI)				0.3103				0.3034
Absent	421 (49%)	144 (51%)	277 (47%)		419 (48%)	194 (51%)	225 (47%)	
Present	441(50%)	137 (48%)	304 (52%)		440 (51%)	188 (49%)	252 (52%)	
Unknown	5 (1%)	2 (1%)	3 (1%)		5 (1%)	2 (1%)	3(1%)	
Perineural Invasion (PNI)				<0.0001*				0.0195
Absent	534 (62%)	203 (72%)	331 (57%)		533 (62%)	221 (57%)	312 (65%)	
Present	326 (%)	78 (27%)	248 (42%)		324 (37%)	161 (42%)	163 (34%)	
Unknown	7 (1%)	2 (1%)	5 (1%)		7 (1%)	2 (1%)	5 (1%)	
MMR status				<0.0001*				0.14
MMR-proficient	795 (92%)	245 (87%)	550 (94%)		794 (92%)	347 (90%)	447 (93%)	
MMR-deficient	68 (8%)	38 (13%)	30 (5%)		68 (8%)	36 (9%)	32 (7%)	
NA	4 (0%)	0 (0%)	4 (1%)		2 (0.2%)	1 (0.3%)	1 (0.2%)	

NA: not available. Fisher’s exact test was used when >25% of the cells have expected counts less than 5. The Pearson’s chi-square and Fisher’s exact test did not include the “NA” and “unknown” group. *p < 0.005 is significant and 0.005 < *p* < 0.05 is suggestive evidence.

The density of CD8+ TILs within the tumor microenvironment was significantly higher in the proximal colon (*p* = 0.02), nodal metastasis-negative (pN stage-negative, *p* < 0.0001), early pathological TNM stage (*p* < 0.0001), poor differentiation (*p* < 0.0001), absence of perineural invasion (PNI, *p* < 0.0001), and MMR-deficient (*p* < 0.0001) colon carcinoma patients (Table 1). High tumor CD274 expression has the suggestive associated with poor differentiation (*p* = 0.02) and with an absence of perineural invasion (PNI, *p* = 0.0195).

### Tumor CD274 expression correlates with high CD8+ T-cell infiltration in colon carcinoma

Interestingly, in stage II–III colon carcinoma, a direct correlation between CD274 expression in tumor cells and CD8+ TILs (Table 2) was observed; 160 of 384 (41.9%) CD274-high tumors contained a high number of CD8+ TILs, and the remaining 223 (58.1%) CD274-high tumors had low CD8+ TILs. Only 123 of 480 (25.6%) CD274-low tumors in which immunohistochemistry for tumor infiltrating lymphocytes could be examined had a high number of CD8+ TILs. Therefore, CD8+ infiltration was significantly higher in high CD274-positive tumors than in low CD274-positive tumors (*p* < 0.0001, Table 2). Moreover, the density of CD8+ TILs was significantly correlated with the level of tumor CD274 in both MMR-proficient (p < 0.0001) and MMR-deficient (p = 0.0068) colon carcinoma patients (Table 2).

**Table 2:** Correlation between CD8+ TILs and tumor CD274/PD-L1 in stage II-III colon carcinoma patients (n = 864).

Parameters	Total no.	High tumor CD274/PD-L1		*p* value
		OR	95% CI	
**All cases**				
**CD8+ TILs**	864			<0.0001*
** Low**	581	1		
**High**	283	2.073	1.554–2.766	
**MMR-proficient**				
**CD8+ TILs**	794			<0.0001*
**Low**	549	1		
**High**	245	1.904	1.404–2.582	
**MMR-deficient**				
**CD8+ TILs**	68			0.0049*
**Low**	30	1		
**High**	38	4.332	1.559–12.035	

*p < 0.005 is significant.

### CD274 expression and CD8+ TILs are significantly associated with 5-year DFS and 5-year OS

**Figure 3:**
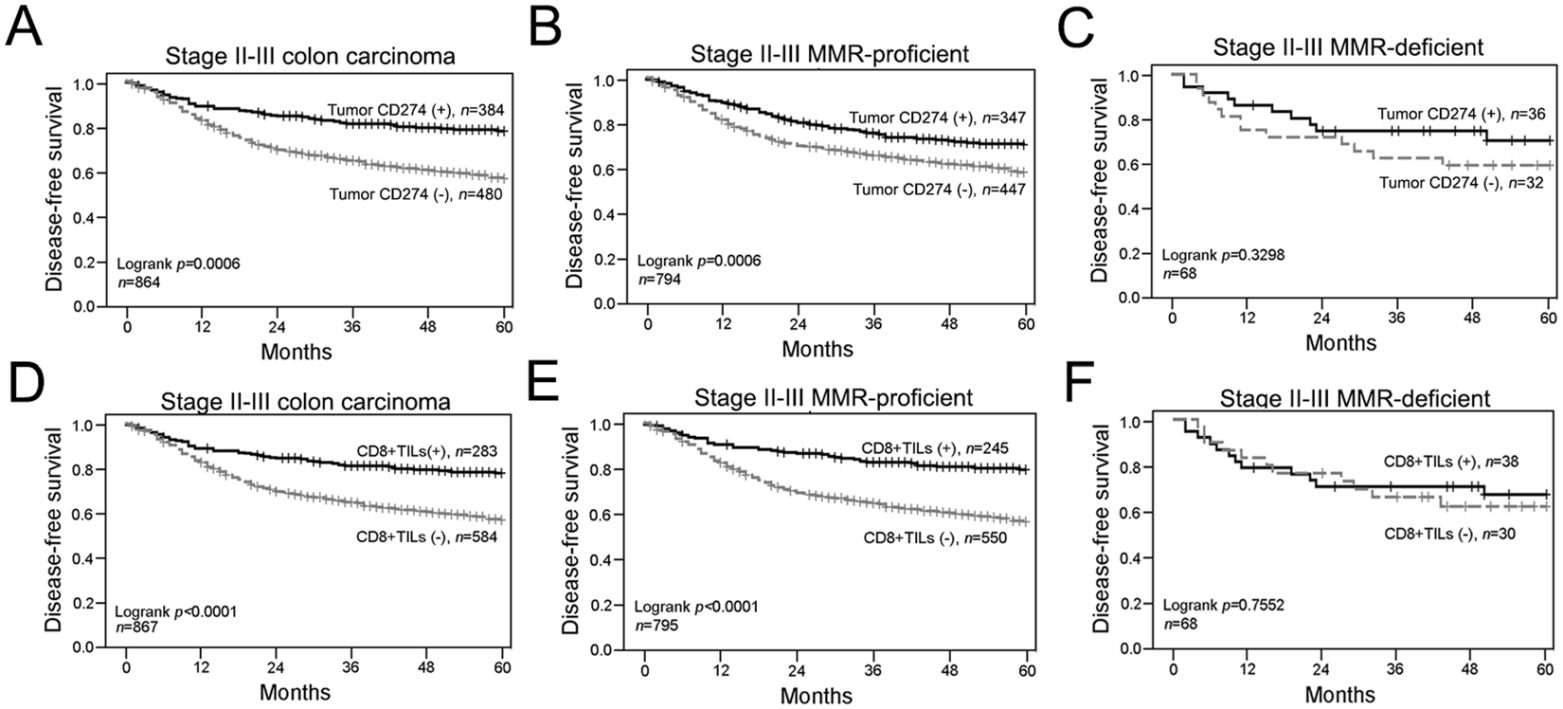
The association of CD8+ TILs and tumor CD274 level in disease-free survival (DFS) among stage II-III colon carcinoma. (**A**) Stage II-III colon carcinoma patients with high tumor CD274 level within the tumor microenvironment had better 5-year DFS (n = 864, *p* = 0.0006). (**B**) High tumor CD274 level in stage II-III MMR-proficient colon carcinoma patients within the tumor microenvironment had improved 5-year DFS (n = 794, *p* = 0.0006). (**C**) In stage II-III MMR-deficient colon carcinoma patients, patients with high tumor CD274 level have no association with 5-year DFS (n = 68, *p* = 0.3298). (**D**) Stage II-III colon carcinoma patients with high density of CD8+ TILs had better 5-year DFS (n = 867, *p* < 0.0001). (**E**) High density of CD8+ TILs in stage II-III MMR-proficient colon carcinoma patients within the tumor microenvironment had improved 5-year DFS (n = 795, *p* < 0.0001). (**F**) In stage II-III MMR-deficient colon carcinoma patients, patients with high CD8+ TILs have no association with 5-year DFS (n = 68, *p* = 0.7552).

For the 5-year follow-up period, the estimated 5-year disease-free survival (DFS) and overall survival (OS) rates were 64% and 71%, respectively (Table 3). By Kaplan-Meier analysis of 5-year DFS, the following factors differed significantly between the two groups: pN stage (negative = 71% vs positive = 56%, p < 0.0001), the level of tumor membranous CD274 expression (high = 70% vs low = 57%, p = 0.0006, Fig. 3A) and the density of CD8+ TILs (high = 78% vs low = 57%, p < 0.0001, Fig. 3D).

**Table 3:** Correlation between clinicopathologic parameters, 5-year DFS and 5-year OS.

Parameters	No^a^	5-yr DFS (%)	*p* value*	5-yr OS (%)	*p* value*
	867	64%		71%	
Sex			0.52		0.39
Female	401	65%		71%	
Male	466	63%		70%	
pT stage			0.02		0.13
pT1–2	32	83%		83%	
pT3–4	833	63%		70%	
pN stage			<0.0001*		<0.0001*
Negative	457	71%		78%	
Positive	410	56%		63%	
Tumor location			0.09		0.02
Distal colon	442	66%		74%	
Proximal colon	425	61%		67%	
Tumor differentiation			0.01		0.02
Well to moderate	734	65%		72%	
Poor	121	54%		62%	
CD8+ TILs			<0.0001*		<0.0001*
High	283	78%		82%	
Low	584	57%		65%	
Tumor CD274/PD-L1			0.0006*		0.0002*
High	384	70%		78%	
Low	480	58%		65%	
CD8+ TILs/Tumor CD274(PD-L1)			<0.0001*		<0.0001*
High/High	160	80%		84%	
High/Low	123	75%		79%	
Low/High	224	63%		73%	
Low/Low	357	53%		60%	
CD8+ TILs and tumor CD274 (PD-L1)			<0.0001*		<0.0001*
High or high	507	71%		78%	
Low/Low	357	53%		60%	

^a^Number of cases may differ due to missing data. *p < 0.005 is significant and 0.005 < *p* < 0.05 is suggestive evidence.

Tumor differentiation (well to moderate = 65% vs poor = 54%, p = 0.01) and pT stage (pT1–2 = 83% vs pT3–4 = 63%, p = 0.02) are also suggestive associated with the risk of 5-yeasr DFS.

In the 5-year OS analysis, patients with pN-negative stage (78% vs 63%, p < 0.0001), distal colon carcinoma (74% vs 67%, p = 0.02) and well to moderate differentiation (72% vs 62%, p = 0.02) had better survival outcome. Moreover, patients with high membranous tumor CD274 level (78% vs 65%, p = 0.0002, Fig. S1A) and high density of CD8+ TILs (82% vs 65%, p < 0.0001, Fig. S1D) had a significantly better survival outcome (Table 3). In addition, tumor PD-L1 and CD8+ TILs were associated with 5-year DFS and 5-year OS in MMR-proficient colon carcinoma (Figs 3B,E, S1B and S1E). However, no significant association between CD8+ TILs and tumor CD274 with 5-year DFS and 5-year OS was found (Figs 3C,F, S1C and S1F).

Next, we assessed the survival differences between groups classified by these two factors. Within the combined group of CD8+ TIL and tumor CD274 subsets, patients with both high CD8+ TILs and tumor CD274 level showed a significantly better DFS (79%) and OS (84%); in contrast, patients with both low CD8+ TILs and tumor CD274 were associated with poorer DFS (53%) and OS (60%). Patients with either high CD8+ TILs or tumor CD274 had similar DFS (75% and 63%) and OS (79% and 73%). These results suggest that combined CD8+ TILs and tumor CD274 expression can be a good prognostic factor for stage II–III colon carcinoma patients.

### Prognostic significance of CD8+ TILs and tumor CD274 expression

In the univariate analysis of 5-year DFS, the following parameters were associated with patient survival rate: sex, age, pT stage, pN stage and tumor location. Moreover, CD8+ TIL counts and tumor CD274 level were statistically associated with 5-year DFS. Patients carrying a low density of CD8+ TILs had an increased risk for a lower 5-year DFS (HR = 2.16, 95% CI = 1.63–2.86, *p* < 0.0001), and those with a low tumor CD274 also had an increased risk for a lower 5-year DFS (HR = 1.51, 95% CI = 1.19–1.91, *p* = 0.0007) compared with patients carrying a high CD8+ TIL count and high tumor CD274 (Table 4). Moreover, patients with both a low CD8+ TIL count and tumor CD274 had a higher risk in terms of 5-year DFS (HR = 1.88, 95% CI = 1.49–2.36, *p* < 0.0001). Similar results were also observed in the univariate analysis of 5-year OS (Table S1). These results indicate that CD8+ TILs and tumor CD274 have significant prognostic value for stage II–III colon carcinoma patients.

**Table 4:** Univariate analysis of DFS and known prognostic factors in stage II-III colon carcinoma patients.

Parameters	No. at risk^a^	Events	HR	95% CI	*p* value
Sex					0.53
Female	401	133	1.00		
Male	466	166	1.08	0.86–1.35	
Age					<0.0001*
<65	387	103	1.00		
≥65	480	195	1.67	1.32–2.12	
pT stage					0.03
T1-2	32	5	1.00		
T3-4	833	293	2.65	1.10–6.42	
pN stage					<0.0001*
Negative	457	125	1.00		
Positive	410	173	1.75	1.39–2.20	
Tumor location					0.09
Proximal colon	425	157	1.00		
Distal colon	442	141	0.82	0.66–1.03	
CD8+ TILs					<0.0001*
High	283	60	1.00		
Low	584	238	2.16	1.63–2.86	
Tumor CD274(PD-L1)					0.0007*
High	384	108	1.00		
Low	480	190	1.51	1.19–1.91	
CD8+ TILs/Tumor CD274 (PD-L1)					<0.0001*
High or high	507	137	1.00		
Low/Low	360	161	1.88	1.49–2.36	

^a^Number of cases may differ due to missing data. *p < 0.005 is significant and 0.005 < *p* < 0.05 is suggestive evidence.

Subsequently, we examined whether the inclusion of other variables significantly associated with stage II–III colon carcinoma survival affected the parameter estimate for CD8+ TILs and tumor CD274 (Table 5). Patients with a low density of CD8+ TILs (HR = 1.86, 95% CI = 1.40–2.49, p < 0.0001) and tumor CD274 level (HR = 1.37, 95% CI = 1.08–1.74, p = 0.009) presented an increased risk for poor DFS (Table 5).

**Table 5:** Multivariate analysis of DFS and known prognostic factors in stage II-III colon carcinoma patients.

Parameters	No. at risk^a^	Events	HR	95% CI	*p* value	HR	95% CI	*p* value
Sex					0.24			0.26
Female	401	133	1.00			1.00		
Male	466	166	1.15	0.91–1.45		1.14	0.91–1.44	
Age					<0.0001*			<0.0001*
<65	387	103	1.00			1.00		
≥65	480	195	1.64	1.29–2.08		1.68	1.32–2.13	
pT stage					0.009			0.007
T1-2	32	5	1.00			1.00		
T3-4	833	227	3.26	1.34–7.94		3.39	1.39–8.26	
pN stage					<0.0001*			<0.0001*
Negative	457	125	1.00			1.00		
Positive	410	173	1.83	1.45–2.31		1.90	1.50–2.40	
Tumor location					0.04			0.06
Proximal colon	425	157	1.00			1.00		
Distal colon	442	141	0.78	0.62–0.98		0.81	0.64–1.01	
CD8+ TILs					<0.0001			
High	283	60	1.00					
Low	584	238	1.86	1.40–2.49		—	—	
Tumor CD274(PD-L1)					0.009			
High	384	108	1.00					
Low	480	190	1.37	1.08–1.74		—	—	
CD8+ TILs and tumor CD274(PD-L1)								<0.0001
High or high	507	137				1.00		
Low/Low	360	161	—	—		1.77	1.41–2.22	

^a^Number of cases may differ due to missing data. *p < 0.005 is significant and 0.005 < *p* < 0.05 is suggestive evidence.

Moreover, patients with either a low density of CD8+ TILs or a low tumor CD274 level within the tumor microenvironment presented an increased risk for poor DFS (HR = 1.77, 95% CI = 1.41–2.22, *p* < 0.0001, Table 5) after adjustment for sex, age, and pT stage, pN stage and tumor location. These results show that the combination of the CD8+ TILs and tumor CD274 level is an independent prognostic factor (Table 5).

## Discussion

The aim of this study was to analyze the interrelationship between tumor CD274, CD8+ TILs and microsatellite instability in a large cohort of patients with colon carcinoma and to evaluate its clinical relevance. Here, we report that CD274 expression is positively correlated with CD8+ T cells infiltration and is associated with a favorable survival outcome in colon carcinoma.

Several different mechanisms have been proposed for CD274 upregulation in tumor cells: (1) innate intrinsic induction: constitutive oncogenic signaling within tumor cells, such as ALK and EGFR, that lead to elevated expression of CD274 (PD-L1)^21,22^; and (2) adaptive immune resistance: induction of CD274 (PD-L1) expression on tumor cells in response to local inflammatory signals produced by active immune response, such as CD8+ cytotoxic T lymphocytes^23,24^. Recent studies have observed the association between CD274 expression and immune cell infiltration within the tumor microenvironment. Interferon-γ (IFN-γ) secreted by the infiltrated CD8+ T lymphocytes was required for CD274 induction, implying that upregulation of CD274 (PD-L1) within the tumor microenvironment served as a negative feedback mechanism, which represents a compensatory immune response by CD8+ T cells and IFN-γ within the tumor microenvironment^4,9^. Furthermore, classification of tumors based on the status of CD274 (PD-L1) and the abundance of TILs has been proposed to be a predictive biomarker for the efficacy of PD-1/PD-L1 immunotherapy: type I (PD-L1+/TIL+; adaptive immune resistance), type II (PD-L1−/TIL−; immunological ignorance), type III (PD-L1+/TIL−; intrinsic induction) and type IV (PD-L1−/TIL+; tolerance)^4,25^. Among these classifications, type I tumors are most likely to benefit from PD-1/PD-L1 immunotherapy, as these tumors have evidence of preexisting intra-tumor T cells that are turned off by PD-L1 engagement. Therefore, being able to correctly define this subset may allow selection of cases that can benefit from PD-1/PD-L1 immunotherapy. Attractively, the type I PD-L1+/TIL+ subset also predicts the therapeutic efficacy of chemotherapy in breast cancer^26–28^, suggesting that the type I PD-L1+/TIL+ subset may not only predict the efficacy of PD-1/PD-L1 immunotherapy but also the response to chemotherapy.

Moreover, chemoradiotherapy has been reported to induce CD274 (PD-L1) upregulation, which is associated with favorable survival in esophageal, bladder and rectal cancer^10,12,13,29,30^. Clinical studies have indicated that chemoradiotherapy synergistically improved PD-1/PD-L1 immunotherapy in tumor control^31–35^. The suspected mechanism is that chemoradiotherapy-induced immune reactions leads to tumor CD274 (PD-L1) upregulation, which will be accessibly targeted by PD-1/PD-L1 immunotherapy^31,32^. Therefore, a deeper understanding of the interactions within the tumor microenvironment and tumor site immune modulation is necessary for further therapeutic approaches. Consistent with these findings, our results showed that tumor CD274 expression and CD8+ TILs within the tumor microenvironment of resected colon carcinoma are associated with favorable 5-year DFS and 5-year OS. Moreover, our results showed that high infiltration of CD8+ TILs is positively correlated with the level of tumor CD274 either in MMR-proficient or MMR-deficient colon carcinoma patients. These results suggested that upregulation of CD274 in colorectal carcinoma is due to an adaptive immune response, based on the association of PD-L1 expression and frequency of CD8+ TILs.

Accumulating evidence indicates that alternations of tumor molecular pathological properties by environmental factors such as diet, nutrients and smoking influence tumor-immune interactions, eventually impacting tumor progression, aggravation and clinical outcome^36–38^. Therefore, the integration of immunological assessment and molecular pathological epidemiology (MPE) within the tumor microenvironment can provide more insights into for the use of therapeutic approaches^37^. Indeed, the increased knowledge of MPE of CRCs has permitted characterization of different molecular subtypes. These advances in MPE will help dissecting the environmental influence on the microenvironment, implementation of personalized therapies and better management of colorectal cancer patients^39,40^. Indeed, colorectal carcinoma patients with MSI-high status showed a high response rate to PD-1/PD-L1 immunotherapy and a good survival rate, suggesting that a higher mutational burden may result in the formation of more tumor antigens (neoantigens) to trigger a robust anti-tumor immune response and to upregulate tumor CD274 expression^41,42^. In addition, our results showed that MMR-deficiency was significantly correlated with a high density of CD8+ TILs but it was not associated with the level of tumor CD274. In our cohort of colon carcinoma, we found that only 8% were MMR-deficient, which is relatively low compared to that in western countries^7^. These results suggest that there are other unknown mechanisms involved in upregulating CD274 expression within the tumor microenvironment in colon carcinoma.

Since there is an inflammatory microenvironment in colorectal cancer, the commensal microbiota influences the intestinal immune system to attract T cell infiltration^43^, which is associated with an improved prognosis in CRC^37,44,45^. On the other hand, recent studies showed that the gut microbiome influences the therapeutic efficacy of PD-1/PD-L1 immunotherapy by increasing the recruitment of T lymphocytes^46,47^. Therefore, as a consequence of this environment, CD274 and CD8+ TILs may be considered to be independent prognostic factors for colon carcinoma. Our study also confirms the link between CD8+ TILs and CD274 expression in stage II–III colon carcinoma and supports the idea that upregulation of CD274 in colorectal carcinoma may be a result of an adaptive immune response, which may have prognostic value and be a therapeutic target for PD-1/PD-L1 immunotherapy.

### Ethical approval

This study was reviewed and approved by the Internal Review Board (IRB) of China Medical University Hospital [Protocol number: CMUH105-REC2-073]. The method was carried out in accordance with the committee’s approved guidelines.

### Informed consent

Informed consents were obtained from all participants in the study.

## Electronic supplementary material

Supplementary information

## References

[1] Hodi FS (2010). Improved survival with ipilimumab in patients with metastatic melanoma. N Engl J Med.

[2] Borghaei H (2015). Nivolumab versus Docetaxel in Advanced Nonsquamous Non-Small-Cell Lung Cancer. N Engl J Med.

[3] Motzer RJ (2015). Nivolumab versus Everolimus in Advanced Renal-Cell Carcinoma. N Engl J Med.

[4] Taube JM (2012). Colocalization of inflammatory response with B7-h1 expression in human melanocytic lesions supports an adaptive resistance mechanism of immune escape. Sci Transl Med.

[5] Francisco LM, Sage PT, Sharpe AH (2010). The PD-1 pathway in tolerance and autoimmunity. Immunol Rev.

[6] Lee LH (2016). Patterns and prognostic relevance of PD-1 and PD-L1 expression in colorectal carcinoma. Mod Pathol.

[7] Droeser RA (2013). Clinical impact of programmed cell death ligand 1 expression in colorectal cancer. Eur J Cancer.

[8] Inaguma S (2016). Clinicopathologic profile, immunophenotype and genotype of CD274 (PD-L1)-positive colorectal carcinomas. Modern Pathology.

[9] Spranger S (2013). Up-regulation of PD-L1, IDO, and T(regs) in the melanoma tumor microenvironment is driven by CD8(+) T cells. Sci Transl Med.

[10] Lim SH (2016). Changes in tumour expression of programmed death-ligand 1 after neoadjuvant concurrent chemoradiotherapy in patients with squamous oesophageal cancer. Eur J Cancer.

[11] Ogura A (2018). Pattern of programmed cell death-ligand 1 expression and CD8-positive T-cell infiltration before and after chemoradiotherapy in rectal cancer. European Journal of Cancer.

[12] Wu CT, Chen WC, Chang YH, Lin WY, Chen MF (2016). The role of PD-L1 in the radiation response and clinical outcome for bladder cancer. Sci Rep.

[13] Hecht M (2016). PD-L1 is upregulated by radiochemotherapy in rectal adenocarcinoma patients and associated with a favourable prognosis. Eur J Cancer.

[14] Li Y (2016). Prognostic impact of programed cell death-1 (PD-1) and PD-ligand 1 (PD-L1) expression in cancer cells and tumor infiltrating lymphocytes in colorectal cancer. Mol Cancer.

[15] Masugi Yohei, Nishihara Reiko, Yang Juhong, Mima Kosuke, da Silva Annacarolina, Shi Yan, Inamura Kentaro, Cao Yin, Song Mingyang, Nowak Jonathan A, Liao Xiaoyun, Nosho Katsuhiko, Chan Andrew T, Giannakis Marios, Bass Adam J, Hodi F Stephen, Freeman Gordon J, Rodig Scott, Fuchs Charles S, Qian Zhi Rong, Ogino Shuji (2016). Tumour CD274 (PD-L1) expression and T cells in colorectal cancer. Gut.

[16] Basile D (2017). Immunotherapy for colorectal cancer: where are we heading?. Expert Opinion on Biological Therapy.

[17] Huang Chih-Yang, Chiang Shu-Fen, Ke Tao-Wei, Chen Tsung-Wei, Lan Yu-Ching, You Ying-Shu, Shiau An-Cheng, Chen William Tzu-Liang, Chao K. S. Clifford (2017). Cytosolic high-mobility group box protein 1 (HMGB1) and/or PD-1+ TILs in the tumor microenvironment may be contributing prognostic biomarkers for patients with locally advanced rectal cancer who have undergone neoadjuvant chemoradiotherapy. Cancer Immunology, Immunotherapy.

[18] Topalian SL (2012). Safety, activity, and immune correlates of anti-PD-1 antibody in cancer. N Engl J Med.

[19] Thompson RH (2006). Tumor B7-H1 is associated with poor prognosis in renal cell carcinoma patients with long-term follow-up. Cancer Res.

[20] Benjamin DJ (2017). Redefine statistical significance. Nature Human Behaviour.

[21] Akbay EA (2013). Activation of the PD-1 pathway contributes to immune escape in EGFR-driven lung tumors. Cancer Discov.

[22] Ota K (2015). Induction of PD-L1 Expression by the EML4-ALK Oncoprotein and Downstream Signaling Pathways in Non-Small Cell Lung Cancer. Clin Cancer Res.

[23] Li Yongshu, Li Fangfei, Jiang Feng, Lv Xiaoqing, Zhang Rongjiang, Lu Aiping, Zhang Ge (2016). A Mini-Review for Cancer Immunotherapy: Molecular Understanding of PD-1/PD-L1 Pathway & Translational Blockade of Immune Checkpoints. International Journal of Molecular Sciences.

[24] Pardoll DM (2012). The blockade of immune checkpoints in cancer immunotherapy. Nat Rev Cancer.

[25] Teng MWL, Ngiow SF, Ribas A, Smyth MJ (2015). Classifying Cancers Based on T-cell Infiltration and PD-L1. Cancer Research.

[26] McLemore, L. E. *et al*. An Immunoscore Using PD-L1, CD68, and Tumor-infiltrating Lymphocytes (TILs) to Predict Response to Neoadjuvant Chemotherapy in Invasive Breast Cancer. *Applied Immunohistochemistry & Molecular Morphology*, **1**, 10.1097/pai.0000000000000485 (2017). 10.1097/PAI.0000000000000485 28422766

[27] Pelekanou, V. *et al*. Effect of neoadjuvant chemotherapy on tumor-infiltrating lymphocytes and PD-L1 expression in breast cancer and its clinical significance. *Breast Cancer Research* **19**, 10.1186/s13058-017-0884-8 (2017). 10.1186/s13058-017-0884-8 PMC5547502 28784153

[28] Kitano A (2017). Tumour-infiltrating lymphocytes are correlated with higher expression levels of PD-1 and PD-L1 in early breast cancer. ESMO Open.

[29] Saigusa S (2016). Implication of programmed cell death ligand 1 expression in tumor recurrence and prognosis in rectal cancer with neoadjuvant chemoradiotherapy. Int J Clin Oncol.

[30] Jomrich G, Silberhumer GR, Marian B, Beer A, Mullauer L (2016). Programmed death-ligand 1 expression in rectal cancer. Eur Surg.

[31] Deng L (2014). Irradiation and anti-PD-L1 treatment synergistically promote antitumor immunity in mice. J Clin Invest.

[32] Dovedi SJ (2014). Acquired resistance to fractionated radiotherapy can be overcome by concurrent PD-L1 blockade. Cancer Res.

[33] Zeng J (2013). Anti-PD-1 blockade and stereotactic radiation produce long-term survival in mice with intracranial gliomas. Int J Radiat Oncol Biol Phys.

[34] Lynch TJ (2012). Ipilimumab in combination with paclitaxel and carboplatin as first-line treatment in stage IIIB/IV non-small-cell lung cancer: results from a randomized, double-blind, multicenter phase II study. J Clin Oncol.

[35] Horinouchi H (2015). Phase I study of ipilimumab in phased combination with paclitaxel and carboplatin in Japanese patients with non-small-cell lung cancer. Invest New Drugs.

[36] Chang, L. C. & Yu, Y. L. Dietary components as epigenetic-regulating agents against cancer. *BioMedicine* **6**, 10.7603/s40681-016-0002-8 (2016). 10.7603/s40681-016-0002-8 PMC4752550 26872811

[37] Ogino S (2018). Integrative analysis of exogenous, endogenous, tumour and immune factors for precision medicine. Gut.

[38] Chen, M. C., Hsu, S. L., Lin, H. & Yang, T. Y. Retinoic acid and cancer treatment. *BioMedicine* **4**, 10.7603/s40681-014-0022-1 (2014). 10.7603/s40681-014-0022-1 PMC4265016 25520935

[39] Dunne PD (2016). Challenging the Cancer Molecular Stratification Dogma: Intratumoral Heterogeneity Undermines Consensus Molecular Subtypes and Potential Diagnostic Value in Colorectal Cancer. Clin Cancer Res.

[40] Thanki K (2017). Consensus Molecular Subtypes of Colorectal Cancer and their Clinical Implications. Int Biol Biomed J.

[41] Rosenbaum MW, Bledsoe JR, Morales-Oyarvide V, Huynh TG, Mino-Kenudson M (2016). PD-L1 expression in colorectal cancer is associated with microsatellite instability, BRAF mutation, medullary morphology and cytotoxic tumor-infiltrating lymphocytes. Modern Pathology.

[42] Llosa NJ (2015). The vigorous immune microenvironment of microsatellite instable colon cancer is balanced by multiple counter-inhibitory checkpoints. Cancer Discov.

[43] Kau AL, Ahern PP, Griffin NW, Goodman AL, Gordon JI (2011). Human nutrition, the gut microbiome and the immune system. Nature.

[44] Ladoire S, Martin F, Ghiringhelli F (2011). Prognostic role of FOXP3+ regulatory T cells infiltrating human carcinomas: the paradox of colorectal cancer. Cancer Immunol Immunother.

[45] Salama P (2009). Tumor-infiltrating FOXP3+ T regulatory cells show strong prognostic significance in colorectal cancer. J Clin Oncol.

[46] Routy B (2018). Gut microbiome influences efficacy of PD-1-based immunotherapy against epithelial tumors. Science.

[47] Gopalakrishnan V (2018). Gut microbiome modulates response to anti-PD-1 immunotherapy in melanoma patients. Science.

