# Supplementary material for: Clinical significance of programmed death 1 ligand-1 (CD274/PD-L1) and intra-tumoral CD8+ T-cell infiltration in stage II–III colorectal cancer
Source: Sci Rep. 2018 Oct 23;8:15658. doi: 10.1038/s41598-018-33927-5 (PMC6199287; doi:10.1038/s41598-018-33927-5)
Supplement: Supplementary file 1 — Supplementary information [file 41598_2018_33927_MOESM1_ESM.doc]

**Clinical significance of programmed death 1 ligand-1 (CD274/PD-L1) and intra-tumoral CD8+ T-cell infiltration in stage II–III colorectal cancer**

Chih-Yang Huang^1,2^, Shu-Fen Chiang^3^, Tao-Wei Ke^4^, Tsung-Wei Chen^5^, Ying-Shu You^2^, William Tzu-Liang Chen^4,*^, K. S. Clifford Chao^3,*^,

^1^Translation Research Core, China Medical University Hospital, China Medical University, Taichung 40402, Taiwan

^2^ Department of nutrition, HungKuang University 43302, Taichung

^3^Cancer Center, China Medical University Hospital, China Medical University, Taichung 40402, Taiwan

^4^Department of Colorectal Surgery, China Medical University Hospital, China Medical University, Taichung 40402, Taiwan

^5^Department of Pathology, China Medical University Hospital, China Medical University, Taichung 40402, Taiwan

*Corresponding author: K. S. Clifford Chao

Cancer Center, China Medical University Hospital, China Medical University, Taichung 40402, Taiwan

E-mail: d94032@mail.cmuh.org.tw

Tel.: 886-4-22052121 ext. 2976

Fax: 886-4-22075011


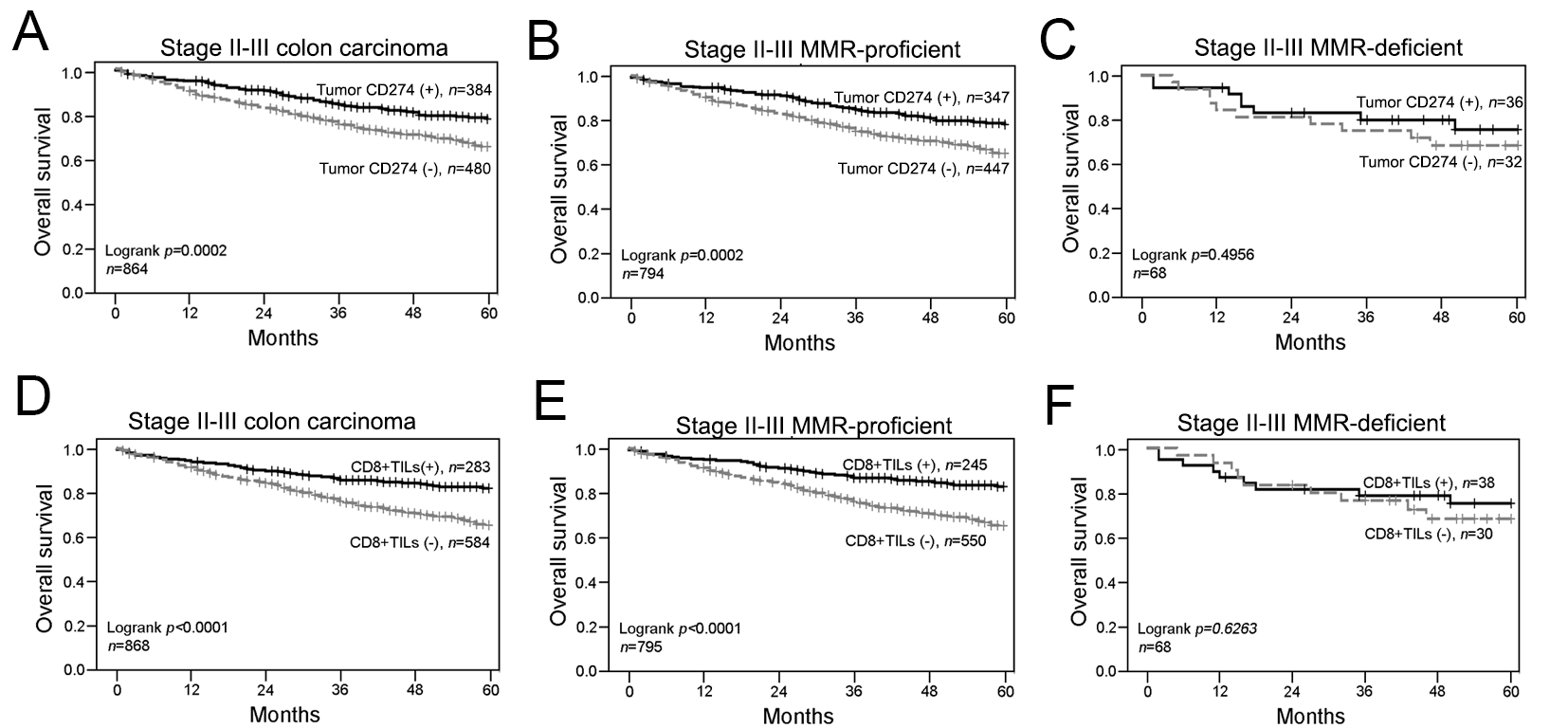


**Figure S1. The association of CD8+TILs and tumor CD274/PD-L1 level in overall survival (OS) among stage II-III colon carcinoma.**

(A) Stage II-III colon carcinoma patients with high tumor CD274/PD-L1 level within the tumor microenvironment had better 5-year OS (n= 864, *p*=0.0002).

(B) High tumor CD274/PD-L1 level in stage II-III MMR-proficient colon carcinoma patients within the tumor microenvironment had improved 5-year OS (n= 794, *p*=0.0002).

(C) In stage II-III MMR-deficient colon carcinoma patients, patients with high tumor CD274/PD-L1 level have no association with 5-year OS (n= 68, *p*=0.4956).

(D) Stage II-III colon carcinoma patients with high density of CD8+TILs had better 5-year OS (n= 867, *p*<0.0001).

(E) High density of CD8+TILs in stage II-III MMR-proficient colon carcinoma patients within the tumor microenvironment had improved 5-year OS (n= 795, *p*<0.0001).

(F) In stage II-III MMR-deficient colon carcinoma patients, patients with high CD8+TILs have no association with 5-year OS (n= 68, *p*=0.6263).

| **Table S1.Univariate analysis of overall survival and known prognostic factors in stage II-III colon carcinoma patients.** | | | | | | | |
| --- | --- | --- | --- | --- | --- | --- | --- |
| **Parameters** | **No. at risk^a^** | **Deaths** | **HR** | **95% CI** | | | ***p* value** |
| Sex |  |  |  |  |  |  | 0.39 |
| Female | 401 | 102 | 1.00 |  |  |  |  |
| Male | 466 | 130 | 1.12 | 0.86 | - | 1.45 |  |
| Age |  |  |  |  |  |  | <0.0001* |
| <65 | 387 | 68 | 1.00 |  |  |  |  |
| ≥65 | 480 | 164 | 2.21 | 1.67 | - | 2.94 |  |
| pT stage |  |  |  |  |  |  | 0.13 |
| T1-2 | 32 | 5 | 1.00 |  |  |  |  |
| T3-4 | 833 | 227 | 1.97 | 0.81 | - | 4.78 |  |
| pN stage |  |  |  |  |  |  | <0.0001* |
| Negative | 457 | 91 | 1.00 |  |  |  |  |
| Positive | 410 | 141 | 1.89 | 1.46 | - | 2.47 |  |
| Tumor location |  |  |  |  |  |  | 0.02 |
| Proximal colon | 425 | 128 | 1.00 |  |  |  |  |
| Distal colon | 442 | 104 | 0.73 | 0.57 | - | 0.95 |  |
| CD8+TILs |  |  |  |  |  |  | <0.0001* |
| High | 283 | 48 | 1.00 |  |  |  |  |
| Low | 584 | 184 | 2.00 | 1.46 | - | 2.75 |  |
| Tumor CD274(PD-L1) |  |  |  |  |  |  | 0.0003* |
| High | 384 | 78 | 1.00 |  |  |  |  |
| Low | 480 | 154 | 1.66 | 1.26 | - | 2.18 |  |
| CD8+TILs/Tumor CD274 (PD-L1) |  |  |  |  |  |  | <0.0001* |
| High or high | 507 | 102 | 1.00 |  |  |  |  |
| Low/Low | 357 | 130 | 1.95 | 1.51 | - | 2.53 |  |
| ^a^Number of cases may differ due to missing data. **p*<0.005 is significant and 0.005<*p*<0.05 is suggestive evidence. | | | | | | | |

| **Table S2.Multivariate analysis of overall survival and known prognostic factors in stage II-III colon carcinoma patients.** | | | | | | | | | | | | | |
| --- | --- | --- | --- | --- | --- | --- | --- | --- | --- | --- | --- | --- | --- |
| **Parameters** | **No. at risk^a^** | **Deaths** | **HR** | **95% CI** | | | ***p* value** |  | **HR** | **95% CI** | | | ***p* value** |
| Sex |  |  |  |  |  |  | 0.13 |  |  |  |  |  | 0.14 |
| Female | 401 | 102 | 1.00 |  |  |  |  |  | 1.00 |  |  |  |  |
| Male | 466 | 130 | 1.23 | 0.94 | - | 1.59 |  |  | 1.22 | 0.94 | - | 1.59 |  |
| Age |  |  |  |  |  |  | <0.0001* |  |  |  |  |  | <0.0001* |
| <65 | 387 | 68 |  |  |  |  |  |  |  |  |  |  |  |
| ≥65 | 480 | 164 | 2.22 | 1.67 | - | 2.94 |  |  | 2.25 | 1.70 | - | 2.99 |  |
| pT stage |  |  |  |  |  |  | 0.047 |  |  |  |  |  | 0.04 |
| T1-2 | 32 | 5 |  |  |  |  |  |  |  |  |  |  |  |
| T3-4 | 833 | 227 | 2.48 | 1.01 | - | 6.07 |  |  | 2.53 | 1.04 | - | 6.18 |  |
| pN stage |  |  |  |  |  |  | <0.0001* |  |  |  |  |  | <0.0001* |
| Negative | 457 | 91 |  |  |  |  |  |  |  |  |  |  |  |
| Positive | 410 | 141 | 2.02 | 1.55 | - | 2.65 |  |  | 2.06 | 1.58 | - | 2.70 |  |
| Tumor location |  |  |  |  |  |  | 0.008 |  |  |  |  |  | 0.01 |
| Proximal colon | 425 | 128 |  |  |  |  |  |  |  |  |  |  |  |
| Distal colon | 442 | 104 | 0.70 | 0.54 | - | 0.91 |  |  | 0.72 | 0.55 | - | 0.93 |  |
| CD8+TILs |  |  |  |  |  |  | 0.002* |  |  |  |  |  |  |
| High | 283 | 48 |  |  |  |  |  |  |  |  |  |  |  |
| Low | 584 | 184 | 1.68 | 1.21 | - | 2.32 |  |  | - | - |  | - |  |
| Tumor CD274 (PD-L1) |  |  |  |  |  |  | 0.002* |  |  |  |  |  |  |
| High | 384 | 78 |  |  |  |  |  |  |  |  |  |  |  |
| Low | 480 | 154 | 1.54 | 1.17 | - | 2.02 |  |  | - | - |  | - |  |
| CD8+TILs and tumor CD274 (PD-L1) |  |  |  |  |  |  |  |  |  |  |  |  | <0.0001* |
| High or high | 507 | 102 |  |  |  |  |  |  | 1.00 |  |  |  |  |
| Low/Low | 357 | 130 | - | - |  | - |  |  | 1.85 | 1.42 | - | 2.40 |  |
| ^a^Number of cases may differ due to missing data. **p*<0.005 is significant and 0.005<*p*<0.05 is suggestive evidence. | | | | | | | | | | | | | |
